# External ocular surface bacterial isolates and their antimicrobial susceptibility patterns among pre-operative cataract patients at Mulago National Hospital in Kampala, Uganda

**DOI:** 10.1186/1471-2415-13-71

**Published:** 2013-11-15

**Authors:** Barnabas Mshangila, Musana Paddy, Henry Kajumbula, Charles Ateenyi-Agaba, Binta Kahwa, Jeremiah Seni

**Affiliations:** 1 Department of Ophthalmology, Makerere University College of Health Sciences, P.O. Box 7072, Kampala, Uganda; 2 Mbeya Referral Hospital, P.O. Box 419, Mbeya, Tanzania; 3 Department of Medical Microbiology, Makerere University College of Health Sciences, P.O. Box 7072, Kampala, Uganda; 4 Department of Microbiology and Immunology, Catholic University of Health and Allied Sciences Bugando, P.O. Box 1464, Mwanza, Tanzania

**Keywords:** Antimicrobial susceptibility, Cataract patients, Uganda

## Abstract

**Background:**

Endophthalmitis is a severe complication of cataract surgery which leads to high ocular morbidity and visual loss even with antibiotic treatment. Bacterial ocular floras are the implicated causative agents. This study was undertaken to evaluate the external ocular surface bacterial isolates and their antimicrobial susceptibility patterns among pre-operative cataract patients at Mulago National Hospital.

**Methods:**

This cross sectional study enrolled consecutively 131 patients scheduled for routine cataract surgery in the Department of Ophthalmology at Mulago National Hospital in Kampala, Uganda. Eyelid margin and conjunctival swabs were collected and processed using standard microbiological procedures to identify bacterial isolates and their respective antimicrobial susceptibility patterns.

**Results:**

Of 131 patients involved (mean age 63.3 ± 14.5 years), 54.2% (71/131) were females. The eyelid margin and conjunctival samples were culture positive in 59.5% (78/138) and 45.8% (60/138) respectively. The most common organisms identified were Coagulase-negative Staphylococci (CoNS) [65.9% (91/138)] and *Staphylococcus aureus* [21.0% (29/138)]. CoNS showed the highest resistance to tetracycline (58.2%, 53/91) and erythromycin (38.5%, 35/91), whereas in *S. aureus* the resistance to tetracycline and erythromycin were 55.2% (16/29) and 31.0% (9/29) respectively. Methicillin resistant CoNS (MRS) and Methicillin resistance *S. aureus* (MRSA) were 31.9% (29/91) and 27.6% (8/29) respectively. There were low resistance rates for CoNS, *S. aureus* and other bacterial isolates to ciprofloxacin (11.1%-24.2%), gentamicin (5.6-31.0%), tobramycin (17.2% -25.3%) and vancomycin (0.0%).

**Conclusion:**

CoNS and *S. aureus* are the most common bacterial isolates found on the external ocular surface of the pre-operative cataract patients. Ciprofloxacin, gentamicin, tobramycin and vancomycin showed the lowest resistance rates to all bacterial isolates, therefore may be used to reduce bacteria load in the conjunctiva sac among cataract patients prior to surgery.

## Background

Endophthalmitis is an inflammatory condition of the eye often caused by bacterial infection [1,2]. It is a rare but dreaded complication of cataract surgery, as it leads to high ocular morbidity and visual loss even with antibiotic treatment [3].

Most bacteria responsible for postoperative ocular infection are part of the normal microbial flora of the conjunctiva and eyelids of the patients [4,5]. Gram-positive pathogens are responsible for 60% to 80% of acute infections, of which Coagulase-negative Staphylococci (CoNS) are the most frequently isolated pathogens, followed by *Staphylococcus aureus* and *Streptococcus spp*[3,5-7]. Gram-negative organisms are responsible for up to 15% of the infections [7,8]. These bacteria are carried into the eye as surface fluid refluxes through the wound during surgery [4,9]. Also, instruments or intraocular lenses may become contaminated if they touch the ocular surface [9,10].

Use of prophylactic antibiotics in cataract surgery reduces the number of organisms in the conjunctiva and eyelids and thus, reduces the risk of postoperative infection [11-13]. Trends of bacterial resistance have been shown to increase among commonly used antibiotics such as penicillins, erythromycin, and tetracycline [14], however the trend is variable to topical fluoroquinolones, a group of broad-spectrum bactericidal agents most frequently used as pre- and postoperative prophylaxis for ocular surgeries. In some areas the resistance trend is increasing [15-17], whereas in other settings only less than 15% of *S. aureus*, CoNS, *Streptococcus spp* and Gram negative bacteria were resistance to quinolones [7,8,14,18]. Low resistance to vancomycin, cefuroxime and newer quinolones such as ofloxacin, or gatifloxacin has been shown among CoNS, *S. aureus* and *Streptococcus spp*[6,8,13,18,19]. The underlying causes on the increase in antimicrobial resistance are complex and mostly related to interconnected factors related to inherent pathogens’ factors, arbitrary and prolonged use of the drugs and the common practice of self-medication [1,20].

The incidence of postoperative endophthalmitis following cataract surgery in Mulago National Hospital recorded in the year 2010 [2.9% (12/412)] was higher than the findings from studies in different countries where the rates were less than 0.1% [2,13,21-24]. In spite of this relatively higher rate there is no standard evidence based protocol for the choice of antimicrobial agent to prevent post-operative infections in this setting.

Therefore, this study aimed at assessing the external ocular surface bacterial isolates from pre-operative cataract patients’ eyelids and conjunctival samples and their antimicrobial susceptibility patterns at Mulago National Hospital.

## Methods

### Study design and sampling process

This was a hospital based cross-sectional study conducted from September 2011 to March 2012 in the Department of Ophthalmology at Mulago National Hospital. The study involved 131 pre-operative patients who were scheduled for cataract surgery during the study period. Patients with nasolacrimal duct obstruction, prior use of systemic or local antibiotics and/or steroids in the past week, current contact lens wearer and children who required general anesthesia were excluded from the study.

### Data collection and laboratory procedures

The entry point for recruiting patients to participate in this study was during biometry session, whereby, patients who met the inclusion criteria were invited to participate. A thorough explanation on the purpose of the research was provided to all study participants prior to seek for a written informed consent.

External ocular examination using a slit lamp biomicroscope to rule out any focus of infection or inflammation was done thoroughly in all patients and then, demographic data were collected using structured questionnaire.

Ocular swabs were aseptically collected by the principal investigator from patients in the morning on the day of surgery before the application of topical anesthetic, mydriatics, antibiotic or povidone-iodine. Lid margin specimens were collected first followed by conjuntival specimens from the same eye. The patient was asked to look up, and then the lower eyelid margin was swabbed with a sterile cotton swab (Biolab, HUNGARY®) moistened with sterile saline, employing a continuous stroke from the nasal to temporal side and then a second stroke from temporal to nasal side. The inferior conjunctival fornix was swabbed by another sterile cotton swab (Biolab, HUNGARY®), employing the same direction and strokes as for the lid margin without touching eyelid or lashes. The swabs were then inoculated into Brain-heart infusion broth (Biolab®, HUNGARY) and processed in the Clinical Microbiology Laboratory of Makerere University College of Health Sciences using standard operating procedures as follows:

### Culture and identification

Samples incubated in Brain-heart infusion broth overnight were sub-cultured into blood agar, chocolate agar and MacConkey (Biolab®, HUNGARY) agar and incubated at 35-36°C for 24–48 hours. Identification of bacteria was based on conventional microbiological methods. These included Gram stain, hemolytic activity on sheep blood agar, catalase reaction, coagulase reaction, optochin disk test, bacitracin disk test, hippurate hydrolysis and CAMP tests for Gram positive bacteria. For Gram negative bacteria identification was based on colony morphology on blood agar and MacConkey agar and reactions on triple sugar iron, hydrogen sulphide production, indole, motility, citrate, urease and oxidase tests [25].

### Drug susceptibility tests

A standard disc diffusion technique for drug susceptibility test (DST) was performed among all identified isolates as recommended by Clinical and Laboratory Standard Institute (CLSI) [26] on Mueller-Hinton agar (Biolab®, HUNGARY). The following antibiotics which are currently available on the market and are in routine ophthalmic use were tested: Chloramphenicol (30 μg), Gentamycin (10 μg), Tobramycin (10 μg), Oxacillin (1 μg), Polymyxcin-B, Erythromycin (15 μg), Vancomycin (30 μg), Tetracycline (30 μg), Ciprofloxacin (5 μg), and Streptomycin (10 μg) (Biolab®, HUNGARY). Multidrug resistance (MDR) bacteria were defined as isolates which are resistance to three or more classes of drugs [27].

Apart from conventional methods, isolates confirmation and drug susceptibility testing were also done using the Phoenix Automated instrument (Becton-Dickson, Sparks Maryland) as per manufacturer’s instruction.

### Data analysis

Data collected was entered into the computer software (EpiData 3.1), cleaned and analyzed using SPSS 17.1 software according to the study objectives. Continuous variables were described as mean (± standard deviation). Categorical variables were described as proportion and were analyzed to compare the significance of difference in distribution by using Chi square test or Fischer’s exact test where appropriate. The difference in distribution was considered significant if p-value was less than 0.05.

### Quality control

Aseptic techniques were strictly observed during sample collection, transportation and processing. The standard positive and negative reference control strains used were *Staphylococcus aureus* ATCC 25923, *Staphylococcus aureus* ATCC 43300, *Staphylococcus epidermidis* ATCC 12228 and *Escherichia coli* ATCC 25922.

### Study clearance and ethical considerations

Permission to conduct this study was obtained from the Institutional Review Board (IRB) of Makerere University College of Health Sciences and Mulago Hospital Research Committee. A written informed consent was obtained from all study participants. Confidentiality was ensured by giving anonymous codes to the study participants. All protocols and procedures in this study complied with the Declaration of Helsinki.

## Results

A total of 131 pre-operative cataract surgery patients were recruited in the study. The mean age was 63.3 ± 14.5 years (range 23 to 98 years). Of these, 54.2% (71/131) were females. Majority (84.0%, 110/131) of the participants were living in the Central region, followed by Eastern region (11.5%, 15/131), western region (3.8%, 5/131) and only 0.76%, (1/131) were from Northern region. The general educational level of the study population was found to be low with only 23.7% (31/131) having had formal education beyond primary level. More than half of the participants (57.3%, 75/131) had outdoor occupation.

### Eyelid and conjunctival bacterial isolates

Culture was positive in 59.5% (78/131) of the eyelid margin samples and in 45.8% (60/131) of the conjunctival samples. The most common bacterial isolates from the eyelid margin were Coagulase-negative staphylococci 66.7% (52/78) followed by *Staphylococcus aureus* 20.5% (16/78), whereas the respective bacteria accounted for 65% (39/60) and (21.7% (13/60) from conjunctival specimens. Of all the CoNS isolates, *Staphylococcus epidermidis* [76.9% (70/91)] and *Staphylococcus saprophyticus* [18.7% (17/91)] were common. Gram negative bacteria accounted for 10.1% (14/138) from both eyelid and conjuctival swabs (Table 1).

**Table 1:** Proportion of bacterial isolates from eyelid and conjunctival specimens

**Organisms**	**Eyelid**	**Conjunctiva**	**Total**
*Staphylococcus epidermidis*	40 (57.1%)	30 (42.9%)	70 (100.0%)
*Staphylococcus aureus*	16 (55.2%)	13 (44.8%)	29 (100.0%)
*Staphylococcus saprophyticus*	8 (47.1%)	9 (52.9%)	17 (100.0%)
*Streptococcus pneumonia*	1 (25.0%)	3 (75.0%)	4 (100.0%)
Other CoNS^*^	4 (100.0%)	0 (0.0%)	4 (100.0%)
Gram negative rods^**^	9 (64.3%)	5 (35.7%)	14 (100.0%)
Total	78 (56.5%)	60 (43.5%)	138 (100.0%)

^*^Staphylococcus caprae (2), *Staphylococcus hominis* (1) and *Staphylococcus hemolyticus* (1).

^**^Enterobacter cloacae (8), *Proteus mirabilis* (3) and *Acinetobacter spp* (3).

CoNS showed the highest resistance to tetracycline (58.2%, 53/91), followed by erythromycin (38.5%, 35/91), whereas in *S. aureus* the resistance to tetracycline and erythromycin were 55.2% (16/29), 31.0% (9/29) respectively. There were low resistance rates for CoNS, *S. aureus* and other bacterial isolates to ciprofloxacin (11.1%-24.2%), gentamicin (5.6-31.0%), and tobramycin (17.2% - 25.3%). Methicillin resistant CoNS (MRS) and Methicillin resistance *S. aureus* (MRSA) were 31.9% (29/91) and 27.6 (8/29) respectively. All Gram positive bacterial isolates were sensitive to vancomycin (Table 2).

**Table 2:** Antimicrobial susceptibility pattern of eyelid and conjunctiva isolates

**Bacterial isolates**		**Antimicrobial drugs**									
		**Oxa**^**1**^	**Chlr**^**2**^	**Erth**^**3**^	**Gent**^**4**^	**Tetra**^**5**^	**Cipro**^**6**^	**Vanco**^**7**^	**Strep**^**8**^	**Poly**^**9**^	**Tobra**^**10**^
**CoNS, n (%)**	S	62 (68.1)	64 (70.3)	56 (61.5)	72 (79.1)	38 (41.8)	69 (75.8)	91 (100)	61(67.0)	63 (69.2)	68 (74.7)
**N =91**	R	29 (31.9)	27 (29.7)	35 (38.5)	19 (20.9)	53 (58.2)	22 (24.2)	0 (0.0)	30 (33.0)	28 (30.8)	23 (25.3)
***S. aureus*, n (%)**	S	21 (72.4)	21(72.4)	20 (69.0)	20 (69.0)	13 (44.8)	21 (72.4)	29 (100)	28 (96.6)	28 (96.6)	24 (82.8)
**N = 29**	R	8 (27.6)	8 (27.6)	9 (31.0)	9 (31.0)	16 (55.2)	8 (27.6)	0 (0.0)	1 (3.4)	1 (3.4)	5 (17.2)
Others*, n (%)	S	1 (83.3)	10 (55.6)	1 (25.0)	17 (94.4)	15 (83.3)	16 (88.9)	4 (100)	15 (83.3)	14 (77.8)	14 (77.8)
**N = 18**	R	3 (16.7)	8 (44.4)	3 (75.0)	1 (5.6)	3 (16.7)	2 (11.1)	0 (0.0)	3 (16.7)	4 (22.2)	4 (22.2)

CoNS = Coagulase-negative *Staphylococcus*, *Gram negative rods and *S. pneumonia*, N = number of isolates tested, S = Sensitive, R = Resistant, ^1^Oxacilin, ^2^Chloramphenical, ^3^Erythromycin, ^4^Gentamycin, ^5^Tetracycline, ^6^Ciprofloxacin, ^7^Vancomycin, ^8^Streptomycin, ^9^Polymyxcin-B, ^10^Tobramycin. Others*: Erythromycin and Vancomycin were tested in *S. pneumonia* isolate only.

MDR isolates among CoNS, *S. aureus* and other isolates were found to be 39.6% (36/91), 27.6% (8/29) and 16.7% (3/18) respectively. Bacteria isolates (irrespective of species) from eyelid and conjunctiva showed no significant difference in antimicrobial resistance profiles (Table 3).

**Table 3:** Comparison of resistance profiles of eyelid and conjunctival bacterial isolates

**Drugs**	**Eyelid isolates, n (%)**	**Conjunctival isolates, n (%)**	**Chi-2**	**p-value**
	**(N = 78)**	**(N = 60)**		
**Oxacillin**	22 (31.9 )^*^	19 ( 34.6)^**^	0.0979	0.754
**Chloramphenicol**	27 (34.6)	16 (26.7)	0.9989	0.318
**Erythromycin**	31 ( 66.0)^*^	16 (34.0)^**^	3.2611	0.071
**Gentamicin**	17 (21.8)	12 (20.0)	0.0658	0.798
**Tetracycline**	41 (52.6)	31 (51.7)	0.0109	0.917
**Ciprofloxacin**	16 (20.5)	16 (26.7)	0.7210	0.396
**Vancomycin**	0 (0.0)^*^	0 (0.0)^**^	-	-
**Streptomycin**	22 (28.2)	12 (20.0)	1.2297	0.267
**Polymyxcin**	22 (28.2)	11 (18.3)	1.8164	0.178
**Tobramycin**	18 (23.1)	14 (23.3)	0.0013	0.972

^*^N = 69 and ^**^N = 55 (antimicrobial tests involved Gram positive bacteria only).

## Discussion

Of 131 pre-operative patients with cataract recruited in the study, more than three quarter were above 50 years. This finding was similar to other studies [8,28] and is because the prevalence of cataract increases with age. Majority of patients were from the central region, with primary school education and were engaging in outdoor occupation.

Culture positivity of the eyelid margin samples (59.5%) and conjunctival samples (45.8%) were less than the rates obtained from other studies [8,14,29] but relatively higher than another similar study [28], this may be attributable to different culture techniques used in these studies. The finding of more bacterial isolates in the eyelid samples compared to conjunctival samples is due to colonization and recurrent introduction of bacteria from adjacent skin to the eyelid margin, whereas the presence of physical, biochemical and immunologic defensive mechanisms on the conjunctiva tend to clear microbes [30]. Similar to other related studies [8,14,28,29,31,32], Gram positive bacteria were commonly isolated with CoNS and *S. aureus* predominating as opposed to Gram negatives which accounted for less than 10% on both eyelid and conjunctival samples. Of the CoNS isolated in this study, *S. epidermidis* accounted for more than two third whereas other CoNS were leastly isolated. These findings are closely related to other studies [28,29]. It is well known that other bacteria such as *Propionibacterium spp* and *Corynebacterium spp* may be found on the ocular surfaces [1], but none of these were isolated in the present study. CoNS, *S. aureus* and other *Streptococcus spp* (which are usually found as normal flora) have been implicated as potential causes of post-surgical endophthalmitis [3-5,7], thus their identification from pre-operative patients in this study calls for introduction an ongoing surveillance to establish the trend essential for infection control in this setting.

This study found high rates of resistance to erythromycin and tetracycline among CoNS and *S. aureus*, the findings which are similar to other studies [28,31]. These can be due to readily availability of these antibiotics, thus liable to indiscriminate use as well as the common practice of self-medication found in Uganda. Resistance to chloramphenicol among CoNS, *S. aureus* and other isolates were high ranging from 27.6% to 44.4% and there were low resistance rates to ciprofloxacin, gentamicin and tobramycin. Furthermore, the invitro-susceptibility results of gentamicin compared to tobramycin are promising in this setting as gentamicin is readily available and less expensive than tobramycin. These findings have also been shown in other similar studies [8,28]. The proportion of MRS (31.9%) and MRSA (27.6%) among CoNS and *S. aureus* respectively are relatively similar to another study [29] but higher than the rate reported from another study [8], the difference may be attributable to colonization status of the study population in the respective settings. The isolation of MRS and MRSA signify that these patients cannot benefit from β-lactamase inhibitors such as penicillins, cephalosporins, monobactams and carbepenems as pre-operative prophylactic agents. All Gram positive bacterial isolates in this study were susceptible to vancomycin. Other similar studies also have showed profoundly low resistance rates among bacteria to vancomycin [8,14,28,31]. The MDR isolates among CoNS (39.6%), *S. aureus* (27.6%) and other isolates (16.7%) in the present study and another study in the same setting [33] are relatively higher than from other studies [8,28,31], showing a growing problem of MDR and thus, emphasizing the need for ongoing antimicrobial resistance surveillance to influence infection control and prevention in this setting. Similar to another study [29], there was no difference in antimicrobial resistance profiles among bacteria isolates from eyelid and conjunctiva samples. This raising trend in bacteria resistance at Mulago National Hospital and in other regions [15,29,34] can be limited by judicious prophylactic use of antibiotics, drug susceptibility test guided therapy and ensuring continuous antimicrobial resistance surveillance [17,34].

## Conclusion

The most common bacteria found on the external ocular surface of the pre-operative cataract patients at Mulago National Hospital are CoNS and *Staphylococcus aureus*, majority of these showed high resistances to tetracycline and erythromycin. All bacterial isolates showed the highest susceptibility rates to ciprofloxacin, gentamicin, tobramycin and vancomycin, and therefore these antibiotics may be used to reduce bacteria load in the conjunctiva sac among cataract patients prior to surgery and thus prevent postoperative endophthalmitis.

## Competing interests

The authors declare that they have no competing interests.

## Authors’ contributions

Conceived and designed the study: BM, MP, BK and JS. Specimens’ collection: BM. Supervised the study: MP, HK, CA and BK. Analyzed the data: BM and JS. Wrote the manuscript: BM, MP, HK, AT, BK and JS. All authors have read and approved the final manuscript.

## Pre-publication history

The pre-publication history for this paper can be accessed here:

http://www.biomedcentral.com/1471-2415/13/71/prepub
